# The Utility of First-Aid Packets

**Published:** 1898-10

**Authors:** 


### The Utility of First-Aid Packets.

In a report of the medical work at Santiago, made to the surgeon-general by Lieut. Guy C. M. Godfrey, assistant surgeon, United States army, commanding officer of the hospital corps, company F of the first division of the fifth army corps, the writer says that during the battle the first-aid work was very effective and was done mostly by regimental surgeons and their hospital squads. Many dressings were applied by the line officers and soldiers on the firing line, and in some instances by the wounded men themselves. Major S. Q. Robinson, who commanded the Aguadores dressing station on July 1st, says that only about ten patients came there who had not been dressed by by first-aid packets. Words can hardly express the appreciation which the officers and men of the line have for first-aid packets. They realize now as never before the value and importance of instruction in first-aid work. The very small number of suppurating wounds can readily be accounted for by the prompt application of these dressings.—*New York Medical Record.*

